# One step forward towards deep‐learning protein complex structure prediction by precise multiple sequence alignment construction

**DOI:** 10.1002/ctm2.1689

**Published:** 2024-06-16

**Authors:** Wei Zheng, Qiqige Wuyun, Yang Zhang

**Affiliations:** ^1^ Department of Computational Medicine and Bioinformatics University of Michigan Ann Arbor Michigan USA; ^2^ Department of Computer Science and Engineering Michigan State University East Lansing Michigan USA; ^3^ Cancer Science Institute of Singapore National University of Singapore Singapore Singapore; ^4^ Department of Computer Science, School of Computing National University of Singapore Singapore Singapore; ^5^ Department of Biochemistry, Yong Loo Lin School of Medicine National University of Singapore Singapore Singapore

**Keywords:** antibody−antigen interactions, deep‐learning, multiple sequence alignments, protein−protein complex structure prediction

1

Proteins are the ‘workhorse’ molecules carrying out nearly all biological functions within living organisms. Most of the functions of a protein are performed through its interactions with other proteins, known as protein−protein interactions (PPIs). Common instances include the interactions between antibodies and antigens, which bolster an organism's capacity to identify and combat external pathogens, the binding of ligand and receptor proteins that instigate cellular signalling cascades, and the interplay between enzyme and substrate proteins that facilitate metabolic processes. The important functional roles played by PPIs render them pivotal targets in numerous contemporary drug design initiatives.^1^

Significant efforts have been made to determine the three‐dimensional structures of PPI complexes, which could provide a geometric and physical landscape to facilitate biological function annotation and drug discovery efforts targeting PPIs. Although structural biology techniques such as X‐ray crystallography and Cryo‐Electron Microscopy offer the most precise structural insights into PPIs, they frequently demand significant resources and lack scalability for resolving molecular structures across the entire proteome. The most recent success of artificial intelligence (AI) algorithms, such as AlphaFold2,^2^, ^3^ has enabled the computational prediction of protein structures with remarkable accuracy, opening avenues for obtaining high‐quality structures of proteins and PPI complexes through computational means.

Most AI methods train models from large‐scale experimental structures built on co‐evolutionary information obtained from multiple sequence alignments (MSAs), as the latter can offer critical structural information of the target protein. Specifically, during evolution when a mutation occurs at one residue site and disrupts its interaction with other residues, the protein may become unstable, making it difficult for species with such mutation to survive. However, if the interacting residues mutate at the same time and stabilize the protein structure, the species can continue to survive. This phenomenon refers to ‘co‐evolution’. Since proteins in current organisms have all undergone the rigours of co‐evolution over hundreds of millions of years, aligning a vast array of protein sequences in MSAs can effectively deduce information about protein spatial distances between residues (see Figure 1A). Although MSA and the co‐evolutionary information have been successfully utilized by various AI‐based 3D structure prediction approaches,^2^, ^4^ due to the lack of large‐scale PPI sequence databases, however, constructing comprehensive MSAs and deducing reliable quaternary co‐evolutionary structure insights remain a major bottle‐neck problem for PPI complex structure predictions.

**FIGURE 1:**
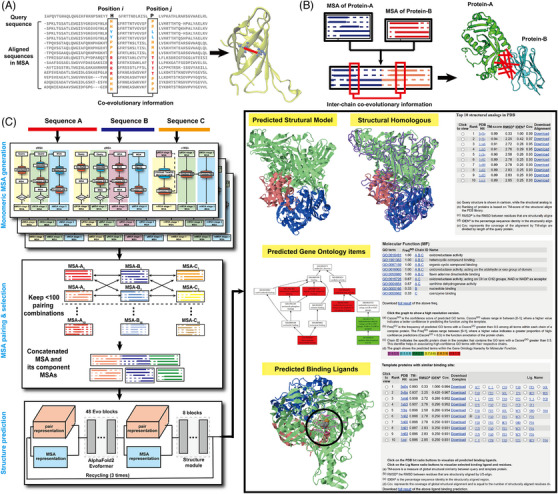
The DMFold pipeline for protein complex structure prediction. (A) Co‐evolutionary information from monomer MSAs contains 3D structural insights for protein structure prediction. (B) Co‐evolution from appropriately paired protein complex MSAs can be used for protein complex structure prediction. (C) DMFold pipeline for protein complex structure prediction followed by structure‐based function annotations.

To tackle the challenge, we developed the DMFold^5^, ^6^ algorithm, in which we utilized a simple protocol to construct PPI MSAs and deduce inter‐chain co‐evolutionary information by pairing monomer‐chain MSAs that can benefit from abundant sequence resources from metagenome repositories^7^ (see Figure 1B). As outlined in Figure 1C, DMFold first constructs monomer MSAs for each component chain through iterative dynamic programming and Hidden Markov Model searches against multiple metagenome sequence libraries.^8^ To ensure orthologous sequence alignments, which is critical for inter‐chain co‐evolutionary map deduction, DMFold selectively links the homologous sequences of different chains coming from the same species, where a new AI‐driven MSA scoring strategy has been introduced to rank the MSAs prior to sequence pairing. The assembled PPI MSAs are subsequently fed into an end‐to‐end deep‐learning neural network module to generate PPI complex structure predictions. To enhance the biomedical usefulness, the online DMFold server (https://zhanggroup.org/DMFold/) creates multiple outputs from primary sequences in addition to the PPI complex structure models, including the top 10 experimental structures closest to the target and structure‐based functional annotations on gene ontology, enzyme commission and binding ligands, respectively.

The Critical Assessment of protein Structure Prediction (CASP) is a community‐wide blind experiment which holds biennially and aims at providing an objective benchmark of the state‐of‐the‐art structure prediction technologies. DMFold participated (as ‘Zheng’) in the most recent CASP15 and ranked as the best protein complex structure prediction method by the official CASP assessors,^9^ where the overall Z‐score of DMFold (35.4) was 18% higher than the second best method (29.9) and 2.9 times higher than that of the standard version of AlphaFold2 (12.3) (Figure 2A).

**FIGURE 2:**
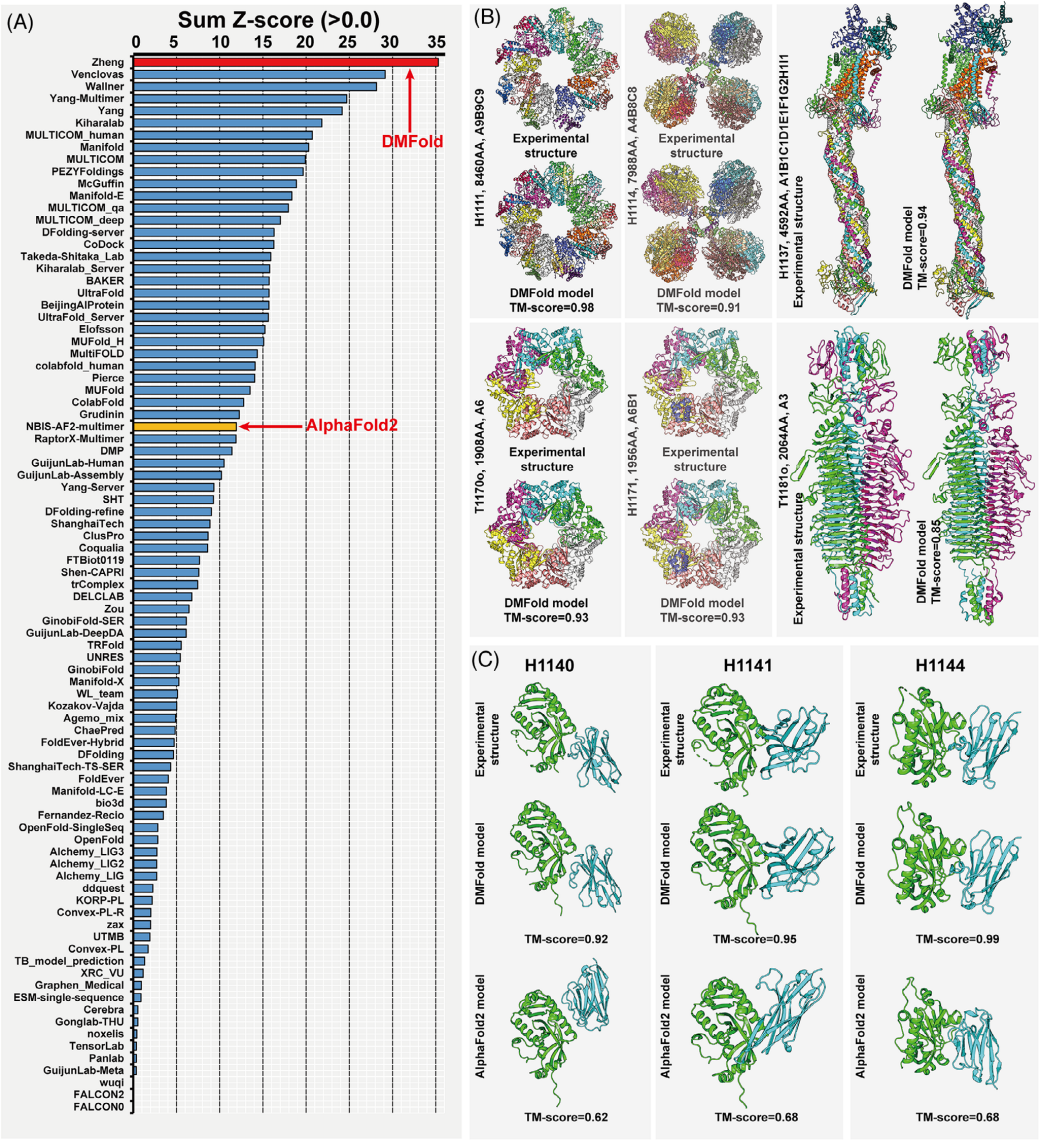
Blind test results of DMFold in CASP15 experiment. (A) Overall ranking of protein complex structure prediction methods based on 41 targets for the 87 registered CASP15 assembly groups, with data taken from the CASP15 official webpage (https://predictioncenter.org/casp15/zscores_multimer.cgi). DMFold (registered as ‘Zheng’) and the public March 2022 version of the AlphaFold2 server (registered as ‘NBIS‐AF2‐multimer’) are marked in red and orange, respectively. (B) DMFold models associated with experimentally solved structures for six large‐size CASP15 complex targets (> 1500 residues) successfully modelled by DMFold. (C) The experimental structures and PPI models predicted by AlphaFold2 and DMFold for three CASP15 nanobody‐antigen complexes (H1140, H1141 and H1144).

Figure 2B lists the results of DMFold on six large‐size protein complexes containing > 1500 residues, where TM‐scores of the DMFold models are .98, .91, .94, .93, .93 and .85, respectively. Here, TM‐score is a standard measure of structure prediction accuracy with larger values indicating higher prediction accuracy, where a TM‐score > .9 generally corresponds to models with medium‐to‐high experimental structure accuracy.^10^ Notably, the two largest targets (H1111 and H1114) contain > 7900 residues both being heteromeric complexes with stoichiometry variable of ‘A9B9C9’ and ‘A4B8C8’, respectively. These results underscore the remarkable capacity of DMFold to model large‐size PPI complexes, towards the solution of a longstanding challenge faced by traditional quaternary structure modelling approaches.

A particularly exciting application of DMFold is on modelling antibody‐ or nanobody‐antigen complexes, a type of PPI that plays pivotal roles in the defence of our body against the invasion of external pathogens. Figure 2C presents a comparison of structural models by DMFold and AlphaFold2 on three nanobody‐antigen targets in CASP15, which represent three typical interaction modes of nanobodies with mouse CNPase. While AlphaFold2 failed to model the PPI orientations with TM‐score below .7 for all three complexes, DMFold demonstrates exceptional prediction power with three models achieving TM‐scores of .92, .95 and .99, respectively. The capability to accurately model functional PPIs such as antibody−antigen complex structures can help significantly expedite the development process of antibody‐based therapeutics.

In summary, we developed DMFold, an AI‐based pipeline for atomic‐level PPI structure prediction. The major advancement of DMFold lies in the construction of precise multi‐chain MSAs by iteratively collecting and pairing high‐quality monomeric MSAs from huge metagenome sequence databases, which allows for the derivation of reliable cross‐chain co‐evolutionary information and, therefore, enables AI network models to derive accurate quaternary structural patterns and 3D PPI conformations. We expect that the improved power of AI‐based complex structure predictions will significantly enhance the accuracy of large‐scale protein function annotations and the effectiveness of structure‐based drug discovery processes targeting various PPI‐related human diseases.

## AUTHOR CONTRIBUTIONS

Wei Zheng and Qiqige Wuyun wrote the initial version of the manuscript and prepared the figures. Yang Zhang revised and submitted the manuscript.

## CONFLICT OF INTEREST STATEMENT

The authors declare no competing interests.

## ETHICS STATEMENT

Not applicable.
